# Imperforate Hymen Causing Bilateral Hydroureteronephrosis in an Infant with Bicornuate Uterus

**DOI:** 10.1155/2012/102683

**Published:** 2012-06-07

**Authors:** Ayse Secil Eksioglu, Hasim Ata Maden, Gokce Cinar, Yasemin Tasci Yildiz

**Affiliations:** ^1^Department of Pediatric Radiology, Dr. Sami Ulus Women and Children's Hospital, Babür Caddesi, No. 44, Altındag, 06080 Ankara, Turkey; ^2^Department of Pediatric Surgery, Dr. Sami Ulus Women and Children's Hospital, Babür Caddesi, No. 44, Altındag, 06080 Ankara, Turkey

## Abstract

A rare case of imperforate hymen associated with bicornuate uterus in an infant is presented as a cause of bilateral hydroureteronephrosis and pelvic mass in infancy. The importance of postoperative radiologic evaluation for diagnosis of accompanying uterine abnormalities is introduced. A 8-month-old girl with restlessness and intermittent fever was brought to the daily outpatient clinic by her parents. Ultrasound exam showed bilateral grade 4 hydroureteronephrosis and a large cystic pelvic mass. Magnetic resonance scan of the pelvis revealed marked hematocolpos. A cruciate incision was made over the hymen under general anesthesia. During a 6-month followup gradual resolution of bilateral hydroureteronephrosis was documented. Although the details of the uterine anomaly were obscured in preoperative imaging, postoperative US and MR demonstrated bicornuate uterus. Postoperative pelvic radiologic examination is highly recommended to verify the resolution of hematocolpos and to screen for any concomitant anomalies that can have long-term clinical significance.

## 1. Introduction

Imperforate hymen which has an incidence of 0.014%–0.1% is usually asymptomatic until menstruation starts [1–3]. However, under endogenous maternal estrogen stimulation, secretions produced by the fetal uterovaginal mucosa can accumulate in the vagina and uterus resulting in hydrocolpos before puberty. This may cause a mechanical effect on the urethra and bladder and lead to obstructive urinary symptoms. When so, manifestation as a pelvic mass which severely compresses the bladder, and the ureters causing hydroureteronephrosis is rare in infancy [4–6].

 Since imperforate hymen is generally considered not to be associated with other Müllerian abnormalities, further investigation of these patients for concomitant urogenital abnormalities has been thought to be unnecessary until today [7].

 The aim of this report is to increase the awareness about the possibility of this condition as a cause of bilateral hydroureteronephrosis and pelvic mass in infancy and to introduce postoperative radiologic evaluation for diagnosis of accompanying uterine abnormalities. Informed consent was taken from the patient's parents, and the case was presented as a poster in 31st National Radiology Congress, Antalya, Turkey in November 2010.

## 2. Case

 A 8-month-old girl presenting with restlessness and intermittent fever of unknown etiology was brought to the daily clinic by her parents. The parents did not complain about any problems related to urination, and there was some amount of daily urine output.

She had been born term after an uneventful pregnancy via normal vaginal delivery. Far to the parents' knowledge the newborn examination was normal. On physical examination, she had normal vital signs. She was found to have a midline abdominal mass. The rest of the examination was normal. Initial laboratory values were unremarkable, except for a mild leukocytosis and plenty of erythrocytes in the urine. The urine culture did not reveal any pathological findings. Blood urea levels and creatinine levels were normal.

**Figure 1:**
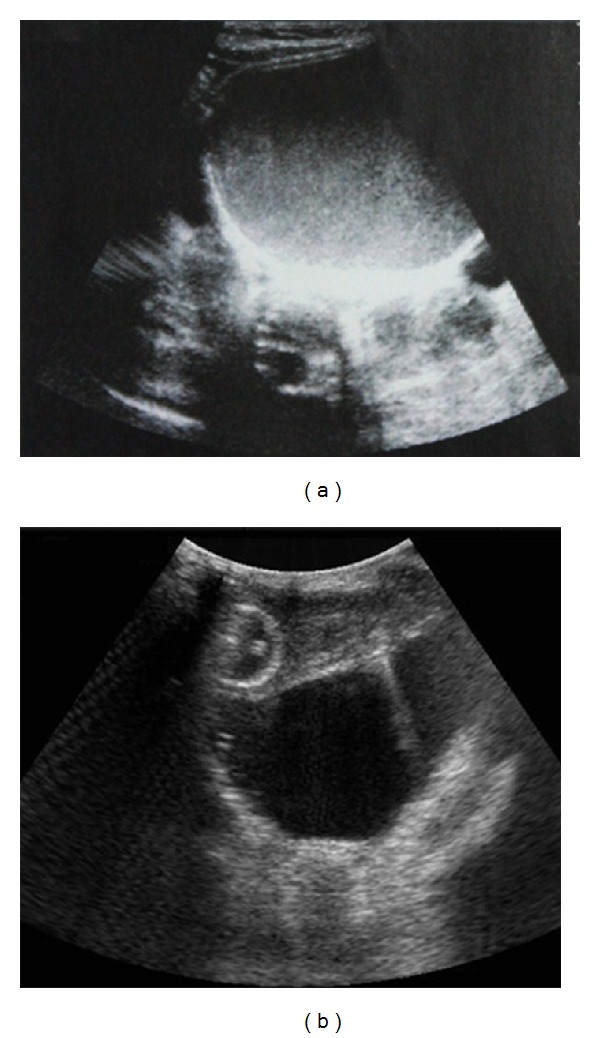
Pelvic US. (a) Transverse view shows a large well-defined cystic mass with internal echoes, which could easily be misinterpreted as overly distended urinary bladder. Note the bilateral ureteral dilatation. (b) Insertion of a Foley catheter makes it clear that the cystic mass is separate from the urinary bladder which is severely compressed and therefore hard to detect on ultrasound.

**Figure 2:**
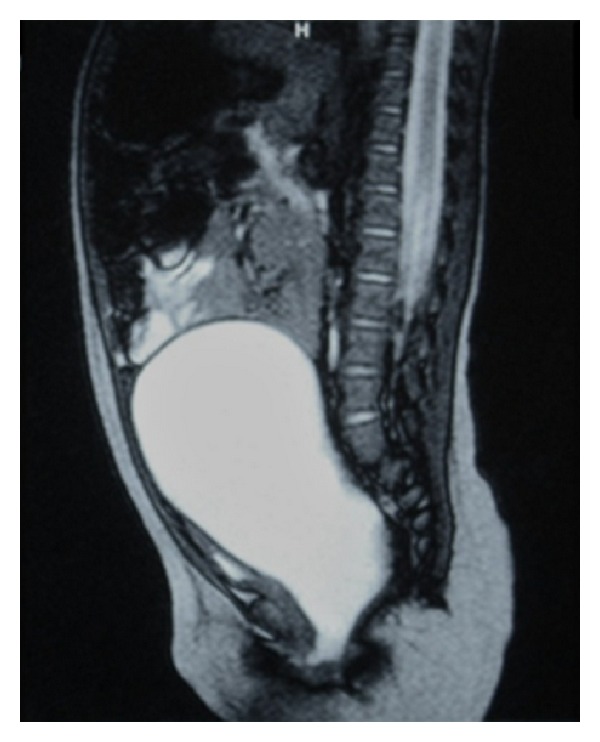
Sagittal-T2-weighted MR image. Marked distention of the uterus and cervix is demonstrated. Note the compressed urinary bladder with little urine in it.

 Abdominopelvic sonography showed bilateral grade 4 hydronephrosis and a large well-circumscribed midline cystic mass including internal echoes. The cyst reached up to the umbilical level (Figure 1(a)). No bladder could be identified on control pelvic ultrasonography (US) exams until the exam was repeated after the insertion of a Foley urethral catheter (Figure 1(b)). Magnetic resonance (MR) scan of the abdomen and pelvis was obtained; it revealed hematocolpos that was causing marked distention of the uterus and cervix. The urinary bladder was significantly compressed (Figure 2). The presumptive diagnosis of hydrometrocolpos secondary to an obstructing lesion was made.

**Figure 3:**
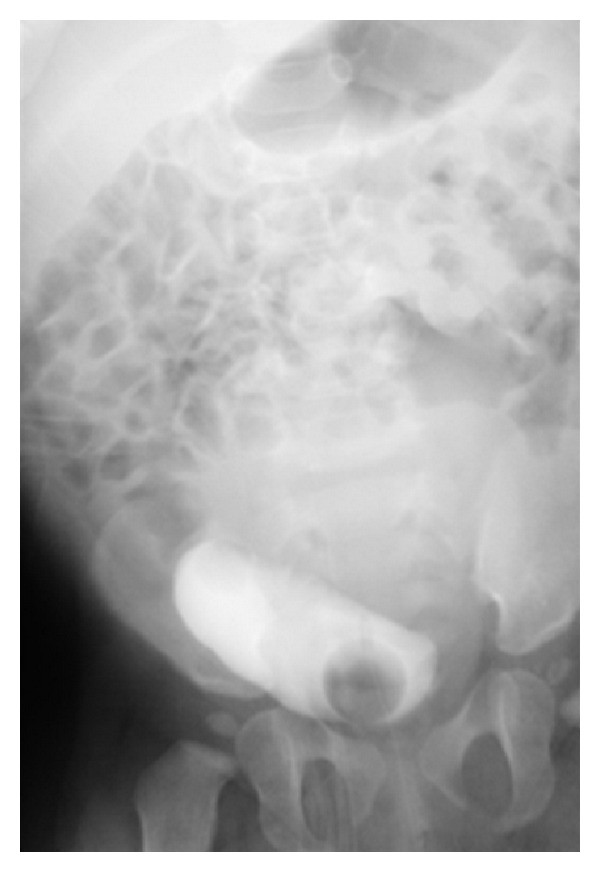
Preoperative VCUG. Image reveals no reflux but a urinary bladder which could not receive appropriate amount of contrast material due to severe compression secondary to hematocolpos.

The patient underwent voiding cystourethrogram (VCUG) which demonstrated no reflux but a compressed urinary bladder with a diminished urine volume of approximately 25cc (Figure 3). The diagnosis of imperforate hymen was made under sedation during the instrumentation for the procedure by the inspection of a protuberant mass on retraction of the labia. The family had not noted any perineal abnormalities prior to presentation to the clinic.

The patient was taken into the operating room, and a simple cruciate incision was made over the hymen under general anesthesia which resulted in drainage of approximately 500 mL cloudy, yellowish, nonbloody mucosal secretions from the vagina. No acute or subacute complications occurred.

**Figure 4:**
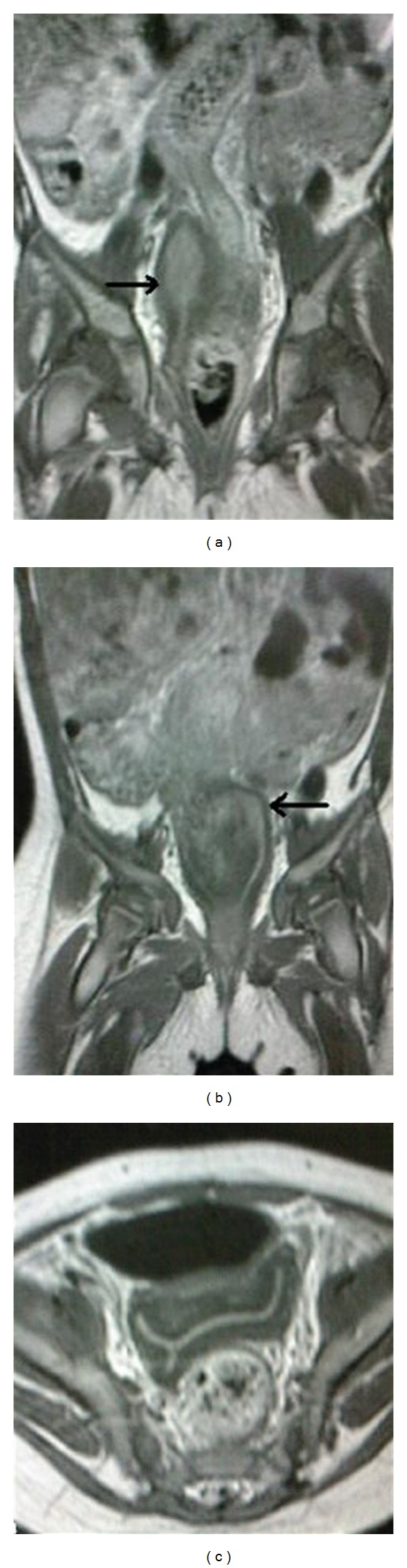
T1-weighed postoperative MR images. (a) and (b): two consecutive pelvic coronal images clearly show the two cavities (arrows) of the uterus separated by an incomplete longitudinal septum which was difficult to depict earlier. (c) axial view through corpus shows bicornuate uterus.

During a period of 6-month followup, repeated ultrasound exams documented the gradual resolution of bilateral hydroureteronephrosis. The suspicion of bicornuate uterus raised by pelvic control ultrasound was verified by a postoperative MR exam (Figures 4(a), 4(b), and 4(c)). 

## 3. Discussion

 Imperforate hymen is an uncommon congenital disorder of the female genital tract [1, 2]. The hymen is an embryological remnant of mesodermal tissue which is supposed to perforate during the later stages of embryonic development [8]. The usual clinical presentation of imperforate hymen is as an expanding abdominal mass and cyclic lower abdominal or back pain in an adolescent girl with primary amenorrhea. It is rarely diagnosed in the neonatal period or infancy. Hydrocolpos or mucocolpos triggered by endogenous maternal estrogen stimulation rarely presents as bilateral severe hydroureteronephrosis in infancy [4–6].

 This particular case did not present as acute urinary retention which would be far more alarming. Less obvious changes in urination can be missed by the family in a child of this age. Imperforate hymen can be hidden under a very nonspecific set of complaints with a broad differential diagnosis, like fever of unknown etiology and restlessness.

In this case ultrasound examination revealed bilateral grade 4 hydronephrosis but was unable to demonstrate the normal pelvic anatomy. It revealed a giant pelvic cystic mass without any change in appearance on more than one ultrasound and which could be easily misinterpreted as a distended urinary bladder since the bladder could not be visualized on either exam. Insertion of a Foley catheter might be helpful to distinguish between a real pelvic mass or urinary bladder overdistension in such cases.

 Incorporation of the external genitalia into the newborn nursery exam and well baby examination is highly recommended so that genital anomalies can be diagnosed early. When the diagnosis of imperforate hymen is made in a newborn or an infant, assuming that there are no urinary signs or obstruction, observation throughout childhood and a planned hymenotomy after the onset of puberty and before menarche is optimal. Surgery in the presence of adequate estrogenization avoids scarring and needs to repeat surgery. When there are signs of urinary obstruction or an abdominal mass as in this case, immediate surgery is needed.

 Whereas imperforate hymen is a problem that could be easily solved by a minor operation without sequela [9], even though rare [7], any accompanying uterine anomaly like bicornuate uterus as in this case could potentially have a long lasting impact on fertility [10].

Early diagnosis of accompanying genital anomalies would not affect the immediate management but would save time and money on the long range. Postoperative imaging is also recommended for the followup of resolution of findings in response to surgery.

Although preoperative US and MR examination both revealed a very distended uterus in the form of a large cystic mass and the details of the uterine anomaly were obscured, postoperative radiologic imaging was diagnostic. Not every case receives pre- and/or postoperative MR exams. The diagnosis is usually based on clinical examination and preoperative ultrasound. No further information regarding the pelvic anatomy may be obtained.

We suggest postoperative radiologic examination, preferably by pelvic ultrasound since it is more accessible and cheaper, both to verify the resolution of hematocolpos and to screen for any concomitant anomalies that can have long-term clinical significance.
